# Oncological and Clinical Impacts of Routine Splenic Flexure Mobilization in Anterior Resection

**DOI:** 10.7759/cureus.74270

**Published:** 2024-11-22

**Authors:** Izna Najam Syed, Mubeen Hasan, Mohammad Badawi, Ben Liu

**Affiliations:** 1 General Surgery, The Royal Wolverhampton NHS Trust, Wolverhampton, GBR; 2 General and Colorectal Surgery, Aston University, Birmingham, GBR; 3 Internal Medicine, Hampshire Hospitals NHS Foundation Trust, Basingstoke, GBR; 4 Colorectal Surgery, The Royal Wolverhampton NHS Trust, Wolverhampton, GBR

**Keywords:** anterior resection, clinical outcomes, colorectal surgery, laparoscopic anterior resection, oncological outcomes, splenic flexure mobilisation

## Abstract

Background

Splenic flexure mobilization (SFM) is widely regarded as one of the most challenging steps in laparoscopic and robotic colorectal surgery, sparking ongoing debate. Some surgeons routinely advocate for SFM, citing its role in achieving greater left colonic reach, which facilitates a safe, tension-free, and well-vascularized anastomosis while adhering to oncological principles. Conversely, others argue that SFM does not consistently ensure these benefits and may increase the risk of complications, including splenic, bowel, or vascular injuries, as well as unnecessarily prolonging the procedure. While traditional surgical textbooks consider SFM a mandatory step in open colorectal resections, limited evidence supports its necessity in minimally invasive approaches.

Aim

This study aims to evaluate whether routinely mobilizing the splenic flexure offers advantages from both oncological and clinical perspectives.

Materials and methods

This retrospective cohort study evaluated the oncological and clinical outcomes of SFM versus splenic flexure preservation (SFP) in anterior resections for malignant pathologies. The study was conducted at New Cross Hospital in Wolverhampton, United Kingdom, over a 24-month period, from March 2022 to March 2024. Anterior resections for benign pathologies were excluded. Data analysis was performed using IBM SPSS Statistics for Windows, Version 24.0 (Released 2016; IBM Corp., Armonk, NY, USA) and Microsoft Excel (Microsoft Corporation, Redmond, WA, USA).

Results

This study included 94 patients, with 65 undergoing SFM and 29 having it preserved (SFP). No significant differences in baseline demographics (age and gender) were observed between the groups. Oncological outcomes revealed a significantly longer median length of resected specimens in the SFM group, although lymph node counts and high vascular ties were comparable between the groups. There were no differences in R0 resection rates. Clinical outcomes showed similar hospital stays and operation durations in both groups. The SFM group had a slightly higher rate of stoma formation but a lower incidence of anastomotic leaks compared to the SFP group. No significant differences in splenic injuries or other complications were noted.

Conclusions

Our study suggests that routine SFM offers certain oncological and clinical benefits. The specimens obtained were more complete for pathological staging. The additional length gained from the maneuver not only results in longer specimens but also provides sufficient mobility of the remaining colon, enabling anastomosis with minimal tension, which helps prevent anastomotic leaks. Surgeons may consider adjusting their practices based on the findings of this study.

## Introduction

Colorectal cancer (CRC) is one of the most prevalent cancers worldwide. According to the GLOBOCAN Index, it ranks third globally and is the fourth leading cause of cancer-related deaths [1]. Most CRC cases are diagnosed in Western countries, where its incidence continues to rise annually. The likelihood of developing CRC is approximately 4-5%, with risk factors including age, chronic disease history, and lifestyle habits [2]. By 2030, the cancer burden is projected to increase by 60%, resulting in over 2.2 million new cases and 1.1 million deaths. Left-sided CRC accounts for 50-60% of all cases, with standard treatment involving complete oncological resection followed by primary anastomosis [1].

Surgical resection is the cornerstone of curative treatment, particularly for cancers of the rectum and sigmoid colon [3]. The success of these surgeries, especially anterior resections, largely depends on achieving clear resection margins [4], minimizing tension on the anastomosis, and preventing complications such as anastomotic leakage [5]. Splenic flexure mobilization (SFM) is considered one of the most challenging aspects of laparoscopic colorectal surgery, and its necessity remains a subject of ongoing debate.

In patients undergoing low anterior resections for rectal cancer, SFM releases the descending and distal transverse colon from their attachments, allowing the descending colon to extend into the pelvis for tension-free anastomosis. SFM also facilitates a longer proximal margin and provides a better vascularized proximal bowel for the anastomosis, as the sigmoid colon is often thick-walled, may have diverticula, and has a poorer blood supply compared to the proximal colon [6,7]. While many surgeons routinely mobilize the splenic flexure as a standard part of the procedure, others argue that selective mobilization is sufficient and that routine SFM may unnecessarily increase operative time and the risk of complications [8].

The primary argument in favor of routine SFM is that it ensures tension-free anastomosis, which is crucial for reducing the risk of anastomotic leakage. Anastomotic leakage not only increases postoperative morbidity and mortality but also significantly impacts long-term oncological outcomes, such as local recurrence rates and overall survival. By routinely mobilizing the splenic flexure, surgeons can ensure that the colon can be brought down to the rectum without tension, potentially reducing the risk of leakage. Furthermore, routine SFM may help achieve more favorable oncological margins, which is particularly important when the tumor is located closer to the rectum [9-11].

The debate over routine versus selective SFM also extends to oncological outcomes. Some studies suggest that routine mobilization may improve oncological outcomes by allowing for more extensive lymph node dissection and clearer resection margins [12], while others have found no significant differences in cancer-specific survival or recurrence rates between patients who undergo routine SFM and those who do not. This raises the question of whether the potential oncological benefits of routine SFM justify the additional surgical risks [8].

Despite its benefits, debates continue regarding the indications for SFM, including when to perform it, the need for additional surgical ports, and technical considerations. SFM is one of the most technically challenging components of colorectal surgery due to its complexity and the splenic flexure’s location in the upper left quadrant, near the spleen and beneath the costal margin [7]. The procedure can be particularly difficult in obese or tall patients, with 0.46-1.4% of patients potentially requiring splenectomy due to splenic injury or bleeding. SFM is also associated with a 10% increase in operative time and may require longer incisions during open surgeries [13]. However, studies have not consistently shown significant benefits in terms of morbidity, oncological outcomes, or survival [6,7,13-15]. Additionally, mastering SFM requires a steep learning curve [1], and the incidence of SFM in surgeries varies between 25% and 60% [14]. Prolonged surgical time has also been linked to an increased risk of postoperative complications, including infections and delayed recovery [8,16]. As a result, some surgeons advocate for a more conservative, case-by-case approach to SFM, reserving it for situations where additional length is required.

Despite the ongoing debate, there is a growing body of evidence that attempts to clarify the clinical and oncological impacts of routine SFM in anterior resections. However, the existing literature is somewhat conflicting, with varying outcomes in terms of operative time, complication rates, anastomotic leakage, and oncological results. The lack of large-scale randomized controlled trials further complicates efforts to draw definitive conclusions.

This study aims to address the debate over whether routine SFM is advantageous from oncological and clinical perspectives. This article was previously presented as a poster abstract at the European Society of Coloproctology (ESCP)’s 19th Scientific and Annual Conference on September 25-27, 2024, and as an oral abstract at the West Midlands Surgical Society’s 2024 conference on November 1, 2024.

## Materials and methods

Study design, duration, and population

This retrospective cohort study was conducted over a 24-month period, from March 2022 to March 2024, at New Cross Hospital in Wolverhampton, United Kingdom. The study focused on patients undergoing anterior resections for malignant pathologies, with data collected retrospectively from clinical records. Both elective and emergency cases were included, encompassing a variety of surgical approaches, including laparoscopic and open techniques.

Inclusion criteria

Our study included non-randomized patients aged 17-95 years who underwent curative anterior resection or low anterior resection for malignant pathologies, with tumors located within 15 cm of the anal margin, as confirmed by preoperative scans during the 24-month period. Both elective and emergency surgeries were included, regardless of the surgical approach (open or laparoscopic).

Exclusion criteria

Patients who underwent anterior resections for benign pathologies, palliative resections, or had incomplete data were excluded from the study.

Operative methods

Anterior resections were performed by Royal College of Surgeons-accredited colorectal consultants. Both elective and most emergency surgeries initially began laparoscopically or robotically, with conversions to open procedures when necessary, although some emergency cases were started as open surgeries. SFM was decided intraoperatively based on the bowel length required to achieve a tension-free, well-perfused anastomosis.

**Figure 1:**
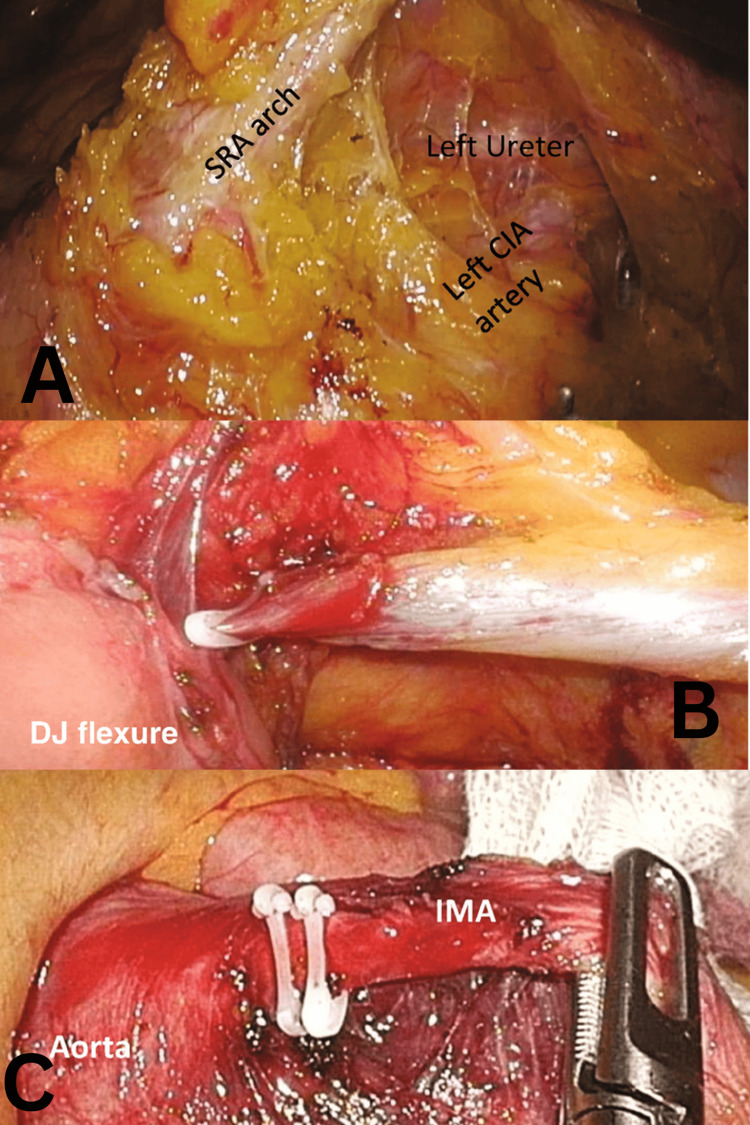
(A) Medial-to-lateral dissection with the SRA arch retracted anteriorly, while the retroperitoneal fascia and structures are swept posteriorly. (B) Ligation of the IMV, lateral to the ligament of Treitz, below the inferior border of the pancreas. (C) Ligation of the IMA, with clips placed proximally at the root, 2 cm distal to the aorta IMA, inferior mesenteric artery; IMV, inferior mesenteric vein; SRA, superior rectal artery Image source: Ng et al. (2023) [17]; Creative Commons Attribution License (CC BY)

The procedure aimed to ensure a tension-free anastomosis with adequate blood supply. The sigmoid colon was retracted, and dissection began at the sacral promontory, progressing toward the ligament of Treitz along an avascular plane beneath the superior rectal artery (Figure 1A). A medial-to-lateral approach was used, with adjustments made for complex cases. Dissection continued to the inferior pancreatic border, with ligation of the inferior mesenteric vein (Figure 1B) and exposure and ligation of the inferior mesenteric artery (IMA) near the aorta (Figure 1C). Care was taken to avoid the marginal artery, and the retrorectal space was identified through further sigmoid retraction.

**Figure 2:**
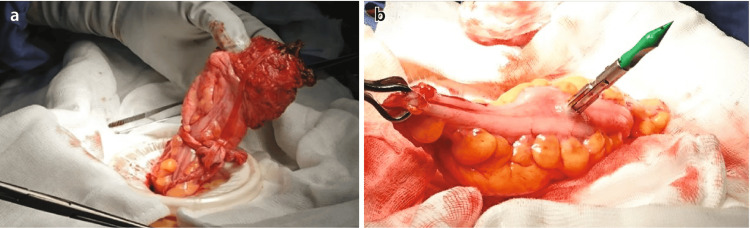
(A) Specimen extraction. (B) Anvil inserted into the antimesenteric border of the colonic conduit for side-to-end anastomosis Image source: Ng et al. (2023) [17]; Creative Commons Attribution License (CC BY)

The rectum was transected using an endoscopic stapler at the confirmed transection level. The mesocolon was divided, and the specimen was exteriorized (Figure 2A, 2B). The colonic mesentery was examined for twisting, and the integrity of the anastomosis was confirmed through visual inspection, air insufflation, and endoscopy. Techniques were adjusted to minimize the risk of low anterior resection syndrome, with temporary stomas created as necessary.

Postoperative care

All patients received care from colorectal clinical nurse specialists who followed the Enhanced Recovery After Surgery (ERAS) guidelines.

Primary outcomes

The study evaluated the oncological quality of the resections, focusing on the length of the resected specimen, the presence of high vascular ties, the total number of lymph nodes retrieved (with particular emphasis on meeting the minimum standard of 12 lymph nodes), and the status of the resection margins. The margins were categorized as R0 (no residual tumor) or R1/R2 (microscopic/macroscopic residual tumor).

Secondary outcomes

Secondary outcomes focused on clinical metrics, including the incidence of anastomotic leaks, length of hospital stay, duration of surgery, splenic injury, stoma formation, and conversion rates to open surgery.

Statistical analysis

Data analysis was conducted using IBM SPSS Statistics for Windows, Version 24.0 (Released 2016; IBM Corp., Armonk, NY, USA). Descriptive statistics were employed to summarize baseline patient characteristics, with continuous variables presented as medians ± SD and categorical variables expressed as percentages. Comparisons between patients undergoing surgery with SFM and splenic flexure preservation (SFP) were made using the chi-square test for categorical variables. To ensure accuracy in cases with small sample sizes, the Fisher’s exact test was used instead of the chi-square test when the predicted frequency in any cell was less than 5. A p-value of <0.05 was considered statistically significant for all analyses.

## Results

This study assessed 104 cases, with 10 excluded according to the exclusion criteria. Of the remaining 94 patients, 65 underwent SFM, while in 29 cases, the splenic flexure was preserved. Table 1 presents the details of tumor staging, the operative approach, and the baseline characteristics of the two groups. The study included patients ranging in age from 17 to 95 years, with a mean age of 56.62 years. No significant baseline differences were observed between the two groups in terms of age and gender.

**Table 1:** Tumor staging, operative approaches, and baseline characteristics in the SFM and SFP groups Test performed: chi-squared test for tumor staging and operative approaches SFM, splenic flexure mobilization; SFP, splenic flexure preservation

Tumor stage	Splenic flexure mobilized (n = 65)	Splenic flexure preserved (n = 29)	p-value	Chi-square value
Staging of tumor (T1/T2/T3/T4)	4/13/42/6	0/1/27/1	0.063	7.29
Staging of tumor (N0/N1/N2)	28/27/10	17/11/1	0.85	0.79
Operative approach				
Open anterior resection	19 (29.2%)	7 (24.13%)	0.73	0.87
Laparoscopic anterior resection	24 (36.92%)	13 (44.82%)	0.64	0.6
Robotic anterior resection	23 (35.38%)	10 (34.4%)	0.87	0.27
Baseline characteristics				
Age (median)	67	72		
Gender	36:29:00	21:08		

When evaluating and comparing the oncological outcomes between the two groups, we found that the group undergoing SFM exhibited a statistically significant increase in the median length of the resected specimen. While the SFM group had higher counts of high vascular ties and lymph nodes greater than 12, these differences were not statistically significant. Additionally, no differences were observed in R0 resection rates between the two groups (Table 2).

**Table 2:** Comparison of oncological outcomes between the SFM and SFP groups Test applied: chi-squared test SFM, splenic flexure mobilization; SFP, splenic flexure preservation

Oncological outcomes	Splenic flexure mobilized (n = 65)	Splenic flexure preserved (n = 29)	p-value	Chi-squared value
Median length (mm)	240	180	0.001	10.82
Presence of high ties (%)	92.5	82.7	0.149	2.07
Median lymph nodes	20	20	0.833	0.044
Significant number of lymph nodes (>12; %)	91	86.2	0.22	1.5
R status (R0; %)	92.3	93.1	0.24	1.3

The clinical outcomes were assessed based on the median length of hospital stay, median duration of surgery, incidence of splenic injuries, need for stoma formation, and occurrence of anastomotic leaks. The median length of hospital stay was similar between the two groups. Although the number of patients requiring stoma formation was higher in the SFM group, this difference was not statistically significant. The group that underwent SFM demonstrated a statistically lower incidence of anastomotic leaks compared to the SFP group (Table 3).

**Table 3:** Comparison of clinical outcomes between the SFM and SFP groups Tests applied: splenic injuries: Fisher’s exact test; remaining variables: chi-squared test SFM, splenic flexure mobilization; SFP, splenic flexure preservation

Clinical outcomes	Splenic flexure mobilized (n = 65)	Splenic flexure preserved (n = 29)	p-value	Chi-squared value/F-value
Median length of hospital stay (days)	7	8	0.88	0.019
Median length of operation (minutes)	264	225	0.72	0.12
Splenic injuries	3	0	0.23	0.82
Stoma formation	6	5	0.882	0.022
Anastomotic leak	2	4	0.0473	3.93

## Discussion

SFM involves detaching the mesocolon from its posterior attachments to the pancreas and Gerota’s fascia along embryological planes, thus exposing the omental bursa [18,19]. This technique allows for the evaluation of retroperitoneal structures and the mobilization of the colon to extend the colonic conduit, ensuring a well-vascularized, tension-free anastomosis [20]. Despite its clear benefits, SFM is a complex and challenging procedure. Some surgeons advocate for its selective use to minimize tension at the colorectal anastomosis [6,15,20]. Currently, there is no consensus on the routine application of SFM in anterior resections.

The use of routine or selective SFM has long been debated among colorectal surgeons [7]. The reported proportion of cases in which SFM is employed varies significantly in the literature. For instance, some centers report an SFM incidence as low as 4% [21], while the 2005-2016 National Surgical Quality Improvement Program (NSQIP) database shows that 41.6% of patients undergo SFM [22], and a survey of 368 colorectal surgeons found that 71.2% routinely mobilized the splenic flexure [23]. In our study, 69.14% of the 94 cases included underwent SFM. This higher rate compared to other studies is likely attributed to the increased number of laparoscopic and robotic anterior resections performed. The variation in the incidence of SFM highlights the absence of clear guidelines for determining which patients require the procedure.

SFM may enhance compliance with pathological metrics, potentially improving oncological outcomes. These metrics include the presence of a high-tie IMA, retrieval of an adequate number of lymph nodes (>12) [24], a longer resected specimen [25], and clear resection margins [26]. Our study found a statistically significant increase in the median length of the resected specimen in the SFM group compared to the No SFM group (240 mm vs. 180 mm, p = 0.001), suggesting that SFM facilitates more extensive resections, potentially improving oncological clearance. For rectal tumors, clear resection margins (R0) of 1 mm are generally considered acceptable [4,27]. In cases of mid-to-distal rectal tumors, where adequate resection margins are crucial for reducing local recurrence, longer oncological specimens like those associated with SFM may be advantageous [26,28]. While our study found that anterior resections with SFM resulted in slightly higher R0 circumferential resection margin rates, the overall difference in R0 margin rates between the SFM and non-SFM groups was not significant. A 2021 meta-analysis by Rondelli et al. [8] comparing perioperative and postoperative outcomes in anterior resections with and without SFM concluded that SFM did not significantly impact R0 margin resection rates. Nevertheless, given the technical advantages of SFM, particularly in complex cases requiring extensive mobilization, this approach may improve the surgeon’s ability to achieve R0 resection and reduce the risk of local recurrence.

In assessing the oncological benefits of SFM in anterior and low anterior resections, the number of lymph nodes retrieved is another important parameter. Our study found that while lymph node retrieval was comparable between the groups (with a median of 20 nodes in both; p = 0.833), the SFM group showed a slightly higher rate of cases achieving a significant lymph node yield (>12 nodes), with 91% in the SFM group compared to 86.2% in the non-SFM group (p = 0.22). These findings align with those of Betge et al. [29], who found that higher lymph node retrieval during CRC resections was associated with better prognostic outcomes in locally advanced CRC (T3/T4). Further studies have shown that suboptimal lymph node retrieval (<12 nodes) in CRC patients is linked to clinically worse outcomes [29,30]. In 2019, Mouw et al. [31] assessed the effect of routine SFM on pathological compliance in patients undergoing low anterior resections for rectal cancer and found that the incidence of inadequate nodal staging was significantly reduced with SFM compared to the non-SFM group. Similarly, Yeo et al. [32] in 2020 determined that harvesting a total of nine or more lymph nodes during rectal cancer resections resulted in significantly better outcomes in patients who underwent neoadjuvant chemoradiotherapy. Therefore, it can be concluded that SFM improves the prognosis of CRC by increasing the number of lymph nodes harvested, ensuring a minimum yield of >12 nodes, which is associated with better oncological outcomes.

As previously mentioned, achieving radical lymph node dissection requires high ligation of the IMA [20,33]. This can lead to ischemia in the distal colon, a complication that can be mitigated by SFM, which lengthens the left colon. Bonnet et al. [34] reported that the length of the proximal colon after a high IMA tie was 10 cm greater than with low vascular ties, allowing for tension-free, well-vascularized anastomosis with lower leak rates. However, Rutegård et al. [35] found no difference in anastomotic leak rates between patients who underwent SFM or had high vascular ties. In contrast, our study found that SFM was associated with significantly lower rates of anastomotic leaks. Overall, our findings support the oncological benefits of SFM, with patients who underwent SFM showing a higher number of high ties and lymph nodes compared to those who had SFP. Furthermore, Beveridge et al. [36] demonstrated in 2018 that high ligation of the IMA during colectomies results in a more straightforward procedure and a higher yield of lymph node retrieval.

One of the clinical outcomes assessed in this study was the median length of hospital stay. Our findings showed that, despite both groups of patients undergoing ERAS, the median length of hospital stay was slightly shorter in the SFM group, although this difference was not statistically significant. This result aligns with a meta-analysis by Rondelli et al. [8], which found that hospital stay was not significantly affected by SFM or preservation during anterior rectal resections. Similarly, studies by Emile et al. [37] and Calleja et al. [38] reported no significant difference in the length of hospital stay between patients who underwent SFM and those who had SFP. These findings suggest that SFM does not significantly prolong the recovery period, and any additional operative time or complexity associated with SFM does not result in longer hospitalization. The comparable hospital stay between groups further suggests that SFM can be performed without placing a substantial burden on healthcare resources in terms of extended postoperative care.

One argument against SFM is the added operative time without significant clinical benefits. Predicting the necessity of performing SFM is important, as our cohort showed that SFM added 39 minutes to the surgical procedure. In 2021, Rondelli et al. [8] found that mobilizing the splenic flexure resulted in significantly longer operative times, without much difference in complication rates or oncological/clinical outcomes compared to SFP. Similar findings were reported by Emile [37], who noted an average of 24.5 additional minutes spent mobilizing the splenic flexure. Meta-analyses by Nowakowski et al. [39] in 2018 and Damin et al. [40] in 2019 also concluded that although SFM added to the overall operative time, it did not result in notable oncological or clinical benefits. However, contrary to these studies, we found that while SFM led to longer operative times, it was not without oncological or outcome-based benefits. Therefore, although SFM requires additional steps to fully mobilize the colon, modestly extending the operative time, the increase in surgical duration is not substantial enough to have a significant clinical impact. This slight increase in time can be considered a reasonable trade-off, given the potential oncological and safety benefits, particularly the lower incidence of anastomotic leaks observed in this group.

Most surgeons who oppose the routine mobilization of the splenic flexure during anterior resections cite the potential risk of splenic injuries. While splenic injuries are a possible complication due to the proximity and manipulation of the spleen during flexure mobilization, the low overall incidence of these injuries suggests that the risk is relatively minimal when performed by experienced surgeons. Moreover, the benefits of reduced anastomotic leak rates may outweigh the slight increase in the risk of splenic injury in select cases where SFM is deemed necessary for achieving tension-free anastomosis and adequate resection margins. In our study, while SFM was associated with a higher incidence of splenic injuries (although statistically insignificant), it also demonstrated significantly lower rates of anastomotic leaks. This suggests that SFM may reduce anastomotic leak rates, potentially by facilitating tension-free anastomosis. Similarly, Rutegård et al. [35] found that while SFM was linked to higher rates of splenic injuries, the incidence of anastomotic leaks was slightly higher with SFP, although this difference was not statistically significant. In contrast, Rondelli et al. [8] concluded that neither the incidence of splenic injuries nor the rate of anastomotic leakage was significantly affected by the mobilization or preservation of the splenic flexure. Given that anastomotic leaks can lead to increased morbidity, longer recovery times, and potentially adverse long-term outcomes, the reduced incidence of these leaks with SFM supports its potential value, despite the associated risk of splenic injury in some cases.

In this study, the rates of stoma formation were similar in both the SFM and SFP groups, with no statistically significant difference (p = 0.882). This suggests that SFM does not significantly increase the likelihood of requiring a stoma. These findings align with the work of Rutegård et al. [35] in 2023, who reported a slightly higher rate of stoma formation in the SFM group in a multicenter cohort study. Similarly, Reddy et al. [41] supported this conclusion, emphasizing that the decision to create a stoma is typically multifactorial, influenced by factors such as patient anatomy, surgical complexity, and the surgeon’s assessment of anastomotic risk, rather than being determined solely by the performance of SFM.

Therefore, while SFM may slightly extend operative time and carry a small risk of splenic injury, these potential drawbacks are outweighed by the significant benefit of reducing anastomotic leak rates. This balance supports the selective use of SFM in cases where it offers oncological and clinical advantages, highlighting the importance of skilled surgical techniques to minimize complications associated with splenic mobilization.

Strengths

The study has several strengths, demonstrating a comprehensive approach to evaluating SFM in anterior resections. Utilizing a robust retrospective cohort design over a two-year period, it incorporates a broad range of patient scenarios, including both elective and emergency surgeries. By comparing key oncological outcomes, such as specimen length and lymph node yield, alongside clinical outcomes like anastomotic leaks and operative duration, the study offers a well-rounded analysis. Additionally, it provides evidence on a debated surgical topic, highlighting the statistically significant reduction in anastomotic leak rates and the increase in resected specimen length with SFM. These findings underscore the potential oncological and clinical benefits of the procedure, offering valuable insights for colorectal surgical practices.

Limitations

The limitations of this study include its retrospective design, potential selection bias, and the relatively short two-year study period, which may limit the generalizability of the findings. Furthermore, as a single-center study relying on clinical records, there is a risk of underreporting minor complications or inconsistencies in the data. Future research involving larger, multicenter cohorts and prospective methodologies would help validate these findings and provide a more comprehensive assessment of the long-term outcomes associated with SFM and non-mobilization techniques.

## Conclusions

SFM appears to be a valuable technique for colorectal resections, offering oncological benefits by increasing resection length and potentially improving lymphatic clearance. Clinically, its association with lower anastomotic leak rates provides compelling support for its routine use in complex rectal resections. Future research with larger cohorts and long-term follow-up is needed to further clarify the impact of SFM on survival outcomes. This may ultimately establish SFM as a standard approach in rectal cancer surgeries, optimizing both oncological and clinical results. Surgeons should consider the findings of this study when evaluating their own practice.
